# Environmental and Economic Impact of Using a Higher Efficiency Ventilator and Vaporizer During Surgery Under General Anesthesia: A Randomized Controlled Prospective Cohort

**DOI:** 10.7759/cureus.39534

**Published:** 2023-05-26

**Authors:** R Ryan Field, Michael-David C Calderon, Steven Mason Ronilo, Michael Ma, Hailey Maxwell, Paulette Mensah, Joseph Rinehart

**Affiliations:** 1 Anesthesiology and Perioperative Medicine, University of California Irvine Health, Orange, USA; 2 Anesthesiology, Kansas City University, Kansas City, USA; 3 Anesthesiology, Stanford Medicine Health Care, Stanford, USA; 4 Anesthesiology, University of California Irvine Health, Orange, USA; 5 Center for Research, University of California Irvine Health, Orange, USA; 6 Anesthesia and Critical Care, University of California Irvine Health, Orange, USA

**Keywords:** low-volume anesthesia machine, environment, sustainability, carbon emissions, aisys cs2, flow-i c20

## Abstract

Background

Compared to traditional breathing circuits, low-volume anesthesia machines utilize a lower-volume breathing circuit paired with needle injection vaporizers that supply volatile agents into the circuit mainly during inspiration. We aimed to assess whether or not low-volume anesthesia machines, such as the Maquet Flow-i C20 anesthesia workstation (MQ), deliver volatile anesthetics more efficiently than traditional anesthesia machines, such as the GE Aisys CS^2^ anesthesia machine (GE), and, secondarily, whether this was in a meaningful economic or environmentally conscious way.

Methodology

Participants enrolled in the study (Institutional Review Board Identifier: 2014-1248) met the following inclusion criteria: 18-65 years old, scheduled for surgery requiring general anesthesia at the University of California Irvine Health, and expected to receive sevoflurane for the duration of the procedure. Exclusion criteria included age <18 years old, a history of chronic obstructive pulmonary disorder, cardiovascular disease, sevoflurane sensitivity, body mass index >30 kg/m^2^, American Society of Anesthesiologists >2, pregnancy, or surgery scheduled <120 minutes. We calculated the total amount of sevoflurane delivered and consumption rates during induction and maintenance periods and compared the groups using one-sided parametric testing (Student’s t-test). There was no suspicion that the low-volume circuit could use more sevoflurane and that the outcome did not answer our research question. One-sided testing allowed for more power to be more certain of smaller differences in our results.

Results

In total, 103 subjects (MQ: n = 52, GE: n = 51) were analyzed. Seven subjects were lost to attrition of different types. Overall, the MQ group consumed significantly less sevoflurane (95.5 ± 49.3 g) compared to the GE group (118.3 ± 62.4 g) (p = 0.043), corresponding to an approximately 20% efficiency improvement in overall agent delivery. When accounting for the fresh gas flow setting, agent concentration, and length of induction, the MQ delivered the volatile agent at a significantly lower rate compared to the GE (7.4 ± 3.2 L/minute vs. 9.1 ± 4.1 L/minute; p = 0.017). Based on these results, we estimate that the MQ can save an estimated average of $239,440 over the expected 10-year machine lifespan. This 20% decrease in CO_2_ equivalent emissions corresponds to 201 metric tons less greenhouse gas emissions over a decade compared to the GE, which is equivalent to 491,760 miles driven by an average passenger vehicle or 219,881 pounds of coal burned.

Conclusions

Overall, our results from this study suggest that the MQ delivers statistically significantly less (~20%) volatile agent during routine elective surgery using a standardized anesthetic protocol and inclusion/exclusion criteria designed to minimize any patient or provider heterogeneity effects on the results. The results demonstrate an opportunity for economic and environmental benefits.

## Introduction

Reduced volatile anesthesia consumption during elective surgery presents an opportunity for economic and environmental impact while delivering patient care. Broadly speaking, the human body takes up a small fraction of the volatile anesthetic agent delivered [1-5], while unused agent eventually vents into the atmosphere as a harmful greenhouse gas. One report calculated that the equivalent impact of anesthetic gases emitted by the researchers’ institution corresponds to about one-third of the climate impact of that facility’s use of electricity and district heating [6]. The type of volatile anesthetic (desflurane, isoflurane, and sevoflurane) used during general anesthesia has an amplified effect on the atmosphere. For example, volatile agents used in standard care, namely, desflurane, isoflurane, and sevoflurane, have a 20-year Global Warming Potential Index (GWP20) of 6,810, 1,800, and 440, respectively [7,8]. These values correspond to the following atmospheric lifetime (years): desflurane: 14, isoflurane: 3.2, and sevoflurane: 1.1. This provoked organizations such as the American Society of Anesthesiologists (ASA) to recommend utilizing low fresh gas flows during the maintenance phase of procedures [5]. Over time, the scientific community has reinforced the legitimacy of these recommendations resulting in a shift from desflurane to sevoflurane from 2012 to 2016 [7]. While sustainable practice changes often increase financial burden initially [9], an efficient volatile delivery system can be a cost saver for medical institutions compared to traditional systems.

Given the complexity of real-world healthcare delivery in an operating room, including consideration of patient safety, we designed a study to practically ask whether or not a low-volume anesthesia machine, such as the Maquet Flow-i C20 (MQ), in an anesthesia machine uses significantly less volatile agent compared to a traditional higher-volume system, such as the GE Aisys CS^2^ (GE). We hypothesized the amount of anesthetic agent delivered by the MQ, under as identical conditions as practically possible, would be significantly lower than that of the GE. We also sought to analyze whether cost differences and environmental impacts had clinical meaning. Testing whether or not the MQ used more volatile anesthetic than the GE device was not of research interest in this study, as it seemed an unlikely outcome conceptually, and would not inform whether or not investing upfront capital in low-volume anesthesia machine designs would be both environmentally friendly and at least budget neutral.

An earlier version of this manuscript was automatically loaded to a preprint server on Research Square on June 15, 2021.

## Materials and methods

With institutional review board (IRB) approval at the University of California Irvine Medical Center (IRB Identifier HS#2014-1248), the research team screened for potential candidates using the surgical schedule from February 2018 to June 2019. The eligibility criteria included subjects 18-65 years old scheduled to receive general anesthesia with sevoflurane. An ineligible subject met the following exclusion criteria at the time of consent: a medical history of chronic obstructive pulmonary disease (COPD), a body mass index (BMI) greater than 30 kg/m^2^, an ASA physical status classification greater than 2, a pregnant female, procedures scheduled less than two hours, a known sensitivity to sevoflurane, a known/suspected susceptibility to malignant hyperthermia, or severe cardiovascular disease with a left ventricular ejection fraction (LVEF) less than 30%. Criteria were selected in an attempt to reasonably homogenize the patient population such that the patients’ differences would not explain any differences in consumption between the two machine types, yield enough case-length data to be confident in our results, and exclude anyone who may have an increased risk of harm from receiving sevoflurane.

Subjects were randomized each recruitment day into either the MQ or GE groups. Randomization, in consideration of patient safety, could not be done upon room entry, as machine checks and plumbing are critical safety tasks to be accomplished before room entry. Therefore, of the eligible patients identified each day, recruitment was randomized by a random number generator to take the next available MQ or GE patient on the surgical schedule, and then to continue with this method of recruitment until the end of each day. Patients were recruited on all weekdays, and all main operating rooms were eligible for recruitment in an effort to allow for unselected case diversity. It was not feasible to blind anesthesia providers and investigators collecting intraoperative data due to the physical differences in machines and the need to interact with the machines throughout the surgical case for patient safety and standard practice. This manuscript was drafted in compliance with the applicable CONSORT guidelines.

Anesthetic management

All subjects received routine preoperative care and surgical preparation, while the research team coordinated with the anesthesia technician team to provide two sevoflurane cassettes/vaporizers after patient recruitment. As the GE only allows one volatile agent cassette to be loaded at a time, the research team exchanged cassettes at incision time for uniform practice to allow potential subgroup analysis and to maintain patient safety. Meanwhile, the MQ allows two vaporizers to be loaded simultaneously and enables switching through the device interface. The study team pre-filled and weighed the two cassettes/vaporizers to avoid disrupting the clinical workflow during the switching process. Additionally, the research team reviewed the study conduct protocol with the anesthesia provider before operating room transport to avoid distraction during patient care and to further ensure understanding and uniform practice between recruitments.

During the induction phase, as defined by the time of room entry to the time of the first incision, the research protocol standardized 15 L/minute of fresh gas flow to match the most commonly noted practice in our institution during this phase of care. We made no study design to work outside of local standards of practice because we felt this would ensure more practical results. Also in line with this aim, we standardized tidal volumes of 6-8 mL/kg ideal body weight, with a positive end-expiratory pressure (PEEP) of 6 centimeters of water (cmH2O). After reaching the steady state phase, defined as the time of first incision to the time of emergence, the anesthesia provider lowered the fresh gas flow to 2 L/minute and the research team switched to the second cassette/vaporizer. During the emergence phase, defined as the time of weaning of anesthesia to patient recovery, the research team continued to log data.

Data collection

The research team collected data manually in real time, as well as collected data exported from the MQ using a USB drive at the end of the case, and, lastly, extracted preoperative data via chart review of the hospital’s electronic medical record. The collected endpoints included age, sex, height, weight, BMI, procedure name, ASA status, randomization (GE or MQ), and significant anesthetic time points (e.g., induction, intubation, weaning, extubation, and Aldrete >8 (a standard index of recovery)). The research team recorded any changes in device settings by the provider and measured values which included ventilator setting, tidal volume (TV), respiratory rate (RR), PEEP, CO_2_ and O_2_ settings (ET/Fi), O_2_%, fresh gas flow, set volume % gas, end-tidal volume %, time of intubation, incision, extubation, the calibration weight, and the anesthetic agent cassette/vaporizer. The research team measured the weight of each gas cassette using a 0.1 g precision scale at three different times, namely, before induction, at the first incision, and after the volatile agent weaning to off. Additionally, a team member tracked the time of intubation (T0), incision (T1), and weaning (T2).

Outcomes and definitions

Our primary outcome evaluated absolute anesthetic consumption during surgical cases between two anesthesia machine types with vaporizer and ventilator technology differences. We measured this outcome in grams of agent consumed. Secondary endpoints included anesthetic consumed during the defined induction and maintenance phases, the length of time to reach Aldrete >8, time to extubation from weaning, and MQ firmware-based volatile anesthetic consumption data.

To calculate consumption, we compared induction (T0-T1), maintenance (T1-T2), and total case (T0-T2) time periods. Secondarily, we normalized the anesthetic consumption data (g consumed/(% anesthetic agent setting • fresh gas glow • time)) to establish a rate of anesthetic consumption, which considered and accounted for necessary and real differences in both anesthetic volume percent settings and time spent in each study phase.

Statistical analysis

With a power of 0.8 (20% type II risk), a significance level of 0.05 (type I risk of 5%), and using a one-sided t-test to compare groups, we calculated two groups of 55 patients (n = 110 total) in each group, MQ or GE, to detect a 25% difference in sevoflurane delivered. Eleven of these N subjects (10% attrition rate) were accounted for in our calculations to address expected unforeseen circumstances leading to an exclusion of a case after recruitment. With final group sizes of 52 and 51, this did indeed occur. Group data are summarized as mean ± standard deviation, or as counts and percentages as appropriate. Parametric testing (Student’s t-test) was performed between groups using Rstudio (www.rproject.org) to compare endpoints.

Based on our calculations and the local cost of sevoflurane during the study period, the MQ must use 25% less sevoflurane to fully replace its acquisition expense during the expected service life. If a machine with different ventilator and vaporizer technologies could pay for itself with cost savings in sevoflurane consumption, we felt this was operationally meaningful, and switching to a potentially more environmentally friendly machine would be at least budget neutral. With a per-case cost of $9.60 ± 5.00 for the conventional group, we calculated 55 subjects in each group to have sufficient power to determine whether or not the MQ group has a cost of $7.20 ± 5.00 per case (assuming equal case variance for this group and staying within 10% or less recruitment attrition).

## Results

Case information

In total, 110 consented patients participated in the study. Seven subjects were excluded due to unforeseen circumstances in the clinical workflow (e.g., a change in anesthetic preference, canceled surgery, etc.) leaving 103 subjects (MQ: n = 52, GE: n = 51) for analysis. Patient and case demographics are shown in Table 1.

**Table 1:** Demographics of enrolled subjects.

N = 103	MQ	GE
Sex		
Female	35	29
Male	17	22
American Society of Anesthesiologists (ASA) status		
ASA 1	6	8
ASA 2	46	43
Age (years)	42 ± 14	44 ± 13
Height (cm)	167 ± 11	169 ± 10
Weight (kg)	71 ± 12	70 ± 13
Body mass index (kg/m^2^)	25 ± 3.0	24 ± 3.1
Total case length (minute)	210 ± 122	236 ± 125
Induction length (minute)	27.0 ± 13.3	35 ± 14.2
Case type		
General	12	18
Gynecology	18	5
Ophthalmology	2	2
Orthopedics	6	12
Otolaryngology	0	1
Plastics	6	7
Urology	8	6

Agent consumption

The MQ consumed significantly less total inhalational anesthetic compared to the GE across the entire case duration (95.5 ± 49.3 vs. 118.3 ± 62.4; p = 0.043) (g). During the induction phase, the MQ used significantly less inhalational anesthetic compared to GE (24.3 ± 12.1 vs. 40.0 ± 34.6; p = 0.003). During the maintenance phase, there was no significant difference between the amount of inhalational anesthetic delivered (71.2 ± 46.9 vs. 78.2 ± 47.0; p = 0.45), although there was a trend favoring MQ.

Rate of inhalational anesthetic delivery

The induction phase was significantly shorter in MQ versus GE cases (Table 1). After calculating the simple consumption rate (gas consumed in grams divided by time delivered), the MQ used less gas compared to the GE machines (0.9 ± 0.4 vs. 1.2 ± 1.0; p = 0.017) (g/minute). There was no significant difference during the maintenance phase between MQ and GE (0.4 ± 0.1 vs. 0.4 ± 0.1; p = 0.79).

We then further added complexity by introducing variables of volume percent anesthetic settings and the actual fresh gas flow rate to the simple consumption rate calculations. During the induction phase, MQ delivered the volatile agent at a significantly lower rate compared to GE (7.4 ± 3.2 vs. 9.1 ± 4.1; p = 0.017). There was no difference during the maintenance phase (7.5 ± 3.1 vs. 7.5 ± 2.1; p = 0.98).

Cost

Our local cost (USD) of inhalational anesthetic per case with MQ was significantly less compared to GE (27.25 ± 14.07 vs. 33.74 ± 17.80; p = 0.043). In other words, surgical procedures performed with MQ were approximately 20% more cost-efficient compared to GE, with all of that cost improvement coming during the induction phase.

Secondary endpoints

There was no significant difference between MQ and GE when evaluating the time (minutes) from weaning to extubation (10.4 ± 6.3 vs. 10.8 ± 6.4; p = 0.74) or recovery milestones, Aldrete score >8 (98.1 ± 51.8 vs. 89.2 ± 52.1; p = 0.38), as seen in Table 2.

**Table 2:** Endpoints summary.

Endpoints	MQ	GE	P-value
Primary endpoints			
Agent delivered (g)			
Induction	24.3 ± 12.1	40.0 ± 34.6	0.003
Maintenance	71.2 ± 46.9	78.2 ± 47.0	0.45
Total	95.5 ± 49.3	118.3 ± 62.4	0.043
Rate delivered (g/% agent*fresh gas flow*minute) (g/minute)			
Induction	7.4 ± 3.2	9.1 ± 4.1	0.017
Maintenance	7.5 ± 3.075	7.5 ± 2.1	0.98
Total agent cost ($)	27.3 ± 14.1	33.7 ± 17.8	0.043
Secondary endpoints			
Wean - extubation (minute)	10.4 ± 6.3	10.8 ± 6.4	0.74
Aldrete >8 (minute)	98.1 ± 51.8	89.2 ± 52.1	0.38

## Discussion

Overall, our results suggest that MQ delivers significantly less (~20%) volatile agent compared to GE during routine elective surgery using a standardized anesthetic protocol. A closer look suggests the distinction is due to the induction period alone; however, it is worth reporting the study was not powered to detect a difference during individual phases, as we only designed for total case impacts. There was a trend toward a reduction in volatile consumption during the maintenance phase. Total case consumption reduction was significant.

Gas flows in the induction phase were kept identical across arms for direct comparison. It is true that we may have described even larger differences if we set both machines to their minimum possible safe gas flows. Fresh gas flows were instead set to the most common local practice and required no change in behavior from the team between arms. Results in the duration of the induction phase were real-world and not influenced by the study protocol. Our total case durations were not significantly different when compared. Rate of consumption testing eliminated any influence from differences in per-case phase times across arms. This metric also included any variance in vaporizer settings.

Certainly, the motivation for our study was to identify if we could be doing more to reduce our perioperative environmental impact, but, realistically, decisions to switch to more environmentally friendly anesthesia machines will not be made if that choice is not cost-effective. Fortunately, a study of economic cost is directly proportional to a reduction in environmental cost. A post-hoc analysis, accounting for the published specific gravity of sevoflurane (1.5203 g/mL) [10], suggests that based on the average cost savings per case, an MQ using only sevoflurane could reasonably replace its acquisition cost within its expected service life. For example, at a median usage of 5.5 cases/day (3.3, 7.8) over five years (3.5, 8.5), it is reasonable to assume the savings may enable an institution to purchase another machine and reduce anesthetic waste in perpetuity. Granted, this cost savings analysis does not account for other maintenance-related costs. Our study did not compare the durability or routine upkeep of these two anesthesia machine types. It also does not factor in the use of volatile agents other than sevoflurane; however, sevoflurane has the least impact on the environment with a midpoint price per bottle. If desflurane were to be used as the sole volatile anesthetic, the MQ would pay for itself after a median caseload of 5.5 cases/day (3.3,7.8) in three years (2.1, 5.1). If desflurane were to be used regardless of the knowledge of its environmental impact, a reduction in cost and environmental impact with this type of anesthesia machine would still occur.

To put sevoflurane usage reduction found in this study in the larger context, over the course of one year in a surgical facility with 20 operating rooms performing 5.5 cases per day, the total difference in greenhouse gas production between the two anesthesia machines would be approximately 402.26 metric tons of CO_2_ [10,11]. This is equivalent to the greenhouse gas production from an average passenger vehicle driven 983,521 miles, the CO_2_ emissions from 48.2 homes’ energy use for one year, or the greenhouse gas emissions avoided by 140 tons of waste recycled instead of landfilled [12].

By extension, the potential for reduced environmental impact is much greater on the national and global scales. Sulbaek Andersen and colleagues estimate that the annual anesthetic emissions in the United States and worldwide are equivalent to CO_2_ emissions of 660,000 metric tons and 4.4 million metric tons, respectively [13]. Theoretically, global adoption of anesthetic-sparing machines could represent a decrease of 127,050 metric tons of CO_2_ within the United States and 847,000 metric tons of CO_2_ globally. This would be equivalent to eliminating the annual CO_2_ emissions of anywhere from 26,975 to 179,830 standard passenger vehicles.

It is important to view the environmental impact of anesthetic agents in the global context of total greenhouse gas emissions. By one estimate, the total contribution of waste anesthetics to climate change is approximately 0.01% of that produced by worldwide fossil fuel combustion [8]. While the impact of inhaled anesthetics may appear small in the global context, it is important to not dismiss them simply because they are medically essential agents. Rather, these data can aid anesthesiologists and surgical institutions in making informed medical and business decisions that are in agreement with their ethical, professional, and environmental values. We also recognize that our protocol speaks to only one facet of environmental impacts as it relates to volatile agent delivery and not a full life cycle assessment of carbon footprint in the larger context. However, our results resonate with the growing support that anesthesia providers should avoid unnecessarily high fresh gas flow rates for all inhaled drugs [1,14] and employ more efficient technologies.

Limitations

We acknowledge several limitations to the study. These include, but are not limited to, the following: a small overall sample size, fluctuations in the concentration of volatile agent setting or fresh gas flow (due to positioning during surgical preparation), and variations in time precision provider charting of the secondary endpoints (weaning, gas off, time in recovery, recovery criteria completion). The protocol procedures were standardized across both devices; however, there are factors such as lack of blinding that the authors could not feasibly account for and should be taken into consideration when drawing conclusions from these data. As previously mentioned, in an ideal world, we could have randomized patients to MQ or GE machines once they already entered the room, but there is not an architectural footprint in a busy operating room to have two machines and hurriedly plumb and check a machine while an often sedated patient waits for surgery and anesthetic induction. Such a construct was deemed too extraordinary to the standard practice of anesthesiology and could result in even grave changes to perioperative outcomes. With our resources, we could only reasonably compare one traditional machine type and one new technology.

## Conclusions

With the growing coalition aimed at addressing climate change, our results support the idea that a newer type of anesthetic delivery system significantly reduced volatile anesthetic agent consumption by 20% compared to a more traditional system. This difference reasonably seems to make both an economic and environmental argument for adoption. Further studies should investigate the impact of implementation on a larger scale. We argue that the modern healthcare team and their medical institutions should be cognizant of the environmental implications of medically necessary care and open to investing in more efficient anesthesia machine technology.
